# Clinical Resilience in Nursing Education: Insights from Thai Instructors on Supporting Student Growth

**DOI:** 10.3390/nursrep15050180

**Published:** 2025-05-20

**Authors:** Pimwalunn Aryuwat, Jessica Holmgren, Margareta Asp, Matanee Radabutr, Annica Lövenmark

**Affiliations:** 1 Boromarajonani College of Nursing Udonthani, Faculty of Nursing, Praboromarajchanok Institute, Udon Thani 41330, Thailand; pimwalunn.aryuwat@bcnu.ac.th; 2 School of Health, Care and Social Welfare, Mälardalen University, 721 23 Vasteras, Sweden; jessica.holmgren@mdu.se (J.H.); margareta.asp@mdu.se (M.A.); annica.lovenmark@mdu.se (A.L.); 3 Boromarajonani College of Nursing Changwat Nonthaburi, Faculty of Nursing, Praboromarajchanok Institute, Nonthaburi 11000, Thailand

**Keywords:** clinical resilience, nursing education, instructors, nursing students, growth, Thailand

## Abstract

**Background:** Resilience is a cornerstone attribute for nursing students, enabling them to adapt to stressful situations encountered during their educational journey and subsequent healthcare career. **Objective:** This qualitative study aimed to explore nursing instructors’ experiences promoting resilience among nursing students during clinical education. **Methods:** Focus groups were conducted with 27 instructors from four nursing colleges in Thailand. Data were analyzed using Braun and Clarke’s inductive thematic analysis approach, guided by the Unitary Caring Science Resilience-Building Model. **Results**: Two main themes emerged: (1) Challenges to Nursing Students’ Resilience and (2) Support Strategies for Enhancing Resilience. Challenges included bridging theory and practice, upholding confidence in clinical skills, adapting to new clinical environments, and managing expectations. Support strategies encompassed providing comprehensive preparation, fostering open communication, implementing peer support systems, and utilizing reflective practice. **Conclusions:** The findings highlight the complex interplay of factors affecting nursing students’ resilience and the multifaceted approaches instructors use to support it. This study underscores the need for a holistic approach to nursing education that addresses clinical competence and psychological well-being. Implications include curriculum redesign to bridge the theory–practice gap, enhanced instructor training in mentorship and resilience-building, implementation of comprehensive student support systems, and technology integration to support learning and resilience.

## 1. Introduction

In the dynamic and challenging field of healthcare, resilience stands as a cornerstone attribute for nursing professionals [1]. Defined as the ability to withstand adversities, adapt to difficulties, make progress in living life, and prepare to deal with new challenges, resilience is particularly critical for nursing students to develop and nurture throughout their educational journey [2]. The intensive and demanding nature of nursing education programs, further compounded by the multifaceted stressors inherent in clinical practice settings, frequently exposes nursing students to elevated levels of stress, anxiety, and emotional exhaustion [1]. These challenges are not merely academic hurdles but significant obstacles that can profoundly impact students’ personal and professional development.

The rigorous nature of nursing education, designed to prepare students for the complexities of modern healthcare, often pushes students to their limits. Clinical rotations, in particular, present a unique set of challenges [3]. Students are thrust into real-world healthcare environments where they must apply theoretical knowledge, develop clinical skills, and navigate complex interpersonal dynamics with patients, families, and healthcare teams. This transition from classroom to clinical setting can be particularly discouraging, as students deal with the realities of patient care, ethical dilemmas, and the emotional toll of working in high-stress environments. As a result of these persistent stressors, a concerning proportion of nursing students grapple with burnout, compromised psychological well-being, and premature attrition from their educational programs [4]. Such experiences not only jeopardize students’ academic performance and professional development but also have profound implications for their overall well-being and future contributions to the nursing profession [5]. The consequences of these challenges extend beyond the individual student, potentially impacting the quality of patient care and exacerbating the ongoing nursing shortage faced by many healthcare systems globally. In this context, fostering resilience emerges as a crucial protective factor in nursing education. Resilience can empower nursing students to constructively engage with challenges, cultivate adaptive coping strategies, and mitigate the adverse psychological effects of stress and adversity [6]. Cultivating resilience empowers students to recover from challenges, maintain optimism when confronted with hardships, and persevere in their academic and career aspirations despite encountering barriers [7].

Resilience-building initiatives in nursing education have been increasingly recognized as essential components of comprehensive student support frameworks [8]. These initiatives help nursing students become more resilient, adaptable, and successful in both their studies and clinical practice by improving nursing students’ skills in handling the challenges and unpredictability of real-world healthcare settings [6]. Fostering resilience enables nursing students to preserve their professional ethics, prioritize patient-focused care principles, and make valuable contributions to their healthcare teams, even when confronted with challenging and high-pressure situations [9]. Nursing instructors occupy a critical position at the intersection of theoretical instruction and hands-on clinical experiences, significantly influencing nursing students’ resilience [10]. The educators serve as clinical instructors, role models, and facilitators of learning. They are uniquely positioned to observe and understand nursing students’ challenges in developing resilience during their clinical education [11]. Nursing instructors play a crucial role in providing support and implementing strategies to foster resilience among nursing students [12].

This paper proposes that the multifaceted role of nursing instructors in promoting student resilience cannot be overstated. Through their pedagogical approaches, instructors can create supportive learning environments encouraging students to reflect on their experiences, develop self-awareness, and build coping strategies [13]. They can provide timely feedback and guidance, helping students to reframe challenges as opportunities for growth and learning. Additionally, nursing instructors can model resilient behaviors and attitudes, demonstrating to students how to navigate the complexities of clinical practice with grace and professionalism [14]. According to standard academic practice, nursing instructors have identified a significant research gap regarding the complex interrelationship between challenges nursing students encounter during clinical placements, the support mechanisms available to them, and the subsequent development of resilience throughout their clinical education journey [15]. Extensive research exists on resilience in nursing students, yet there remains a significant gap regarding the experiences and perspectives of instructors who are instrumental in cultivating resilience among nursing students [14]. This gap in the literature represents a significant opportunity to gain deeper insights into the processes and strategies that contribute to resilience development in nursing education [16].

The absence of research examining nursing instructors’ lived experiences and perspectives represents a significant gap in understanding how clinical education environments contribute to student resilience development, despite instructors’ established influential role in this process [15]. Understanding how instructors perceive and respond to the challenges faced by their students, as well as the strategies they employ to promote resilience, can offer valuable insights for improving nursing education practices and policies. This study aimed to explore nursing instructors’ experiences with challenges and support strategies to promote resilience among nursing students during clinical education. This study will use a qualitative descriptive approach to address the main research question: What are nursing instructors’ experiences in promoting resilience among nursing students during clinical education?

This study is grounded in the Unitary Caring Science Resilience-Building Model by Wei et al. (2021) [17], which provides a robust theoretical foundation integrating Watson’s Human Caring Theory with the philosophy of Unitary Caring Science. This comprehensive framework conceptualizes resilience as a dynamic, evolving process rather than a static trait, particularly relevant for healthcare professionals experiencing prolonged occupational stressors [18]. The model’s transformative approach facilitates a paradigm shift from unidirectional care-giving to a reciprocal energy exchange, acknowledging that healing relationships are fundamentally communal and mutually restorative. Through six interconnected strategies, namely embracing loving-kindness toward self and others, cultivating authentic interpersonal connections, deepening creative self-expression and belonging, harmonizing self-awareness with evolving consciousness, practicing forgiveness to release accumulated negativity, and nurturing sustained faith-hope, the framework offers practical pathways for healthcare professionals to develop resilience while honoring the deeper spiritual dimensions of their caring practice. This theoretical lens enables us to examine how resilience manifests within the complex, intersubjective experience of healthcare delivery during extraordinary challenges.

The theoretical understanding of how resilience is promoted is used as a frame for exploring nursing instructors. This study seeks to make a substantive contribution to the existing body of knowledge on resilience in nursing education by elucidating the experiences and perspectives of nursing instructors through the lens of the Unitary Caring Science Resilience-Building Model. The insights garnered from this research hold the potential to inform and enrich resilience-focused educational initiatives, facilitating the design and implementation of more targeted, evidence-based interventions that are grounded in a holistic, caring-centered approach to resilience. Understanding how resilience is fostered in clinical education settings, as experienced by nursing instructors and interpreted through the Unitary Caring Science framework, can lead to the development of more effective training programs for nursing instructors, enhancing their ability to support student resilience. Additionally, these insights can inform curriculum design, ensuring that resilience-building strategies are integrated throughout the nursing education program in a way that is responsive to the real-world challenges observed by instructors and aligned with the principles of Unitary Caring Science. Additionally, it can also be a tool for nursing students to use in their learning process.

This study represents an important step towards a more comprehensive understanding of resilience promotion in nursing education, viewed through the critical lens of nursing instructors’ experiences and interpreted within the framework of the Unitary Caring Science Resilience-Building Model. Exploring these perspectives seeks to contribute to ongoing efforts to enhance nursing education, supporting the next generation of healthcare professionals in building the resilience needed to thrive in a demanding yet rewarding profession.

## 2. Materials and Methods

### 2.1. Study Design

A qualitative approach was used to obtain comprehensive insights from the participants.

### 2.2. Ethical Considerations

Prior to commencing data collection, ethical approval was obtained from by the Ethics Review Authority of Srimasarakham Nursing College as a representative of the Faculty of Nursing at the Praboromarajchanok Institute, Thailand (approval number 24/2023, approval date 10 November 2023). The study adhered to the principles outlined in the Declaration of Helsinki. All participants received comprehensive information about the study aims, procedures, potential risks and benefits, and their rights as research participants. Written informed consent was obtained from each participant before their involvement in the study. All identifying information was removed from the data, and participants were assigned numerical codes to ensure confidentiality. Digital recordings and transcripts were stored on password-protected devices accessible only to the research team. Data will be securely maintained for five years following study completion, after which it will be permanently deleted in accordance with institutional guidelines. To address potential conflicts of interest, it should be noted that while two authors are employed by the PBRI and work in nursing colleges under the same institutional umbrella, none of the researchers work at any of the four nursing colleges that served as research settings. These two authors work in entirely different nursing colleges from those involved in the study. Additionally, measures were implemented to ensure that the recruitment process and data analysis remained objective and free from undue influence.

### 2.3. Participants

Participants were recruited from four institutions of nursing colleges under the Faculty of Nursing Praboromarajchanok Institute (PBRI) in Thailand. These institutions adhere to a uniform curriculum and operate within a standardized academic framework. The participant group was diverse, consisting of 25 females and 2 males, with ages ranging from 24 to 54 years. Eligibility criteria specified that participants must be full-time nursing instructors with recent clinical supervision experiences of first-year nursing students. The recruitment process involved the solicitation of voluntary participants through institutional gatekeepers at each nursing college. Potential participants were provided with informational sheets detailing the study purpose, procedures, and ethical considerations. Those expressing interest were contacted by the research team to confirm eligibility and schedule focus group sessions. Each individual was assigned a unique identifier using a number coding system (e.g., P1A represents Participant A from focus group 1, P2A represents Participant A from focus group 2, and so on) to maintain participant anonymity.

### 2.4. Data Collection

#### 2.4.1. Focus Group Procedure

Focus group interviews were chosen as the primary data collection method due to their ability to generate rich, qualitative data through group interactions and discussions [19]. This approach allowed for exploring shared experiences and diverse perspectives among the nursing instructors, fostering a deeper understanding of their collective insights into clinical supervision practices. Four digitally facilitated focus group interviews were conducted, one at each participating institution. The digital facilitation of these focus groups enabled broader participation across geographical distances while maintaining the interactive nature of traditional face-to-face focus groups. Each focus group session lasted between 60 and 90 min and was structured with open-ended questions to elicit detailed responses and encourage dialogue among participants.

The primary researcher served as the moderator in all focus group sessions, facilitating discussions and ensuring balanced participation. The moderator guided the discussions to address key research objectives while allowing for the emergence of unanticipated themes [20]. All sessions were digitally recorded with participant permission. Following each focus group, the primary researcher conducted meticulous verbatim transcriptions of the recordings. These transcriptions served as the primary data source for subsequent analysis. The procedural progression for the focus groups was as follows. Initially, research permission requests were dispatched to the directors of the four nursing colleges. Following institutional approval for employing digital focus group methodologies, digital consultations with institutional gatekeepers were convened to elucidate the recruitment and data collection procedures. After gatekeeper endorsement, promotional materials, informational sheets, and consent forms were circulated to potential participants within each institution. Once participants had furnished informed consent, interview schedules were arranged and communicated. Digital platform links were distributed to all gatekeepers, who subsequently disseminated them to participants via various channels, including email and social media platforms. Focus group interviews were then conducted in alignment with predefined guidelines to ensure methodological consistency.

#### 2.4.2. Interview Guide

The interview guidelines (Table 1) were devised with primary and exploratory questions to aid the researcher in adhering to the study’s objectives. These questions encompassed a broad range of nursing instructors’ experiences with nursing students’ challenges and supportive strategies affecting the promotion of resilience during clinical supervision. The credibility and trustworthiness of the methodology were established through three pilot interviews, with participants in these pilot sessions subsequently excluded from the final study sample.

### 2.5. Data Analysis

The analysis process employed was based on Braun and Clarke’s inductive thematic analysis approach, as outlined in their seminal works from 2006 [21] and 2021 [22]. This method was specifically chosen for its ability to facilitate a rigorous and systematic analysis of the collected data, ensuring both data saturation and the meaningful categorization of themes. The focus of this analysis was to explore the challenges and support perceived by nursing instructors related to promoting nursing students’ resilience during their clinical education. The inductive nature of this approach was particularly valuable as it allowed the researchers to delve into the data without preconceived notions or hypotheses. This promoted a high degree of reflexivity among the research team and encouraged an awareness of any inherent biases that might influence the analysis. The researchers ensured a comprehensive and methodical examination of the data by adhering strictly to Braun and Clarke’s six-phase process: Data familiarization involved the primary researcher immersing themselves in the data through repeated readings of the interview transcripts, making initial observations and notes. Initial code generation identified relevant features of the data and coded them systematically across the entire dataset. Theme search organized the generated codes into potential themes that seemed to capture significant patterns in the data. Theme review carefully examined and refined these potential themes to ensure they accurately represented the data and told a coherent story about the experiences of nursing students. Theme definition and naming clearly defined and named each theme to capture its essence and the aspect of the data it represented. Report production created a comprehensive report, supported by relevant data extracts that vividly illustrated each theme. Throughout this process, the primary researcher engaged in regular discussions with all authors. These discussions served as a form of peer review, enhancing the credibility and trustworthiness of the findings. The central focus of the analysis remained firmly on the experiences of nursing instructors regarding challenges and support for promoting nursing students’ resilience during clinical practice, ensuring that the emerging themes were deeply grounded in the data. Data collection continued until saturation was achieved, which occurred after 4 focus group interviews at 4 different nursing colleges.

The data analysis process was meticulous and involved several stages. Initially, the narrative text was decontextualized, isolating it from its original context [23]. This allowed for independent coding and facilitated the generation of categories and themes without being influenced by the surrounding context. Following this, the texts were reevaluated based on the identified code units, ensuring that the coding accurately reflected the content of the interviews. The researchers employed selective text highlighting to aid in theme comparison and the presentation of preliminary findings. This visual technique helped to identify patterns and connections across different interviews and themes. Each theme was explored in depth, with specific quotes carefully selected to emphasize significant aspects of the nursing instructors’ experiences. The coding approach was inductive, allowing themes to emerge naturally from the data rather than fitting the data into predetermined categories. Codes were clustered into significant categories for each participant’s narrative, ensuring that individual experiences were accurately represented. As the analysis progressed, the researchers conducted a reanalysis of the data, which prompted a revision of the initial themes. This iterative process ensured that the final themes were robust and truly representative of the data. The revised findings were then discussed among all authors, and approval was obtained for the final analytical version. This collaborative approach enhanced the validity of the analysis and ensured that multiple perspectives were considered in the interpretation of the data. Throughout the analysis, great attention was paid to the trustworthiness and confirmability of the findings. This was achieved through the use of participant quotations, which were presented under their respective thematic headings. These direct quotes provided evidence for the themes and allowed the voices of the nursing instructors to be heard clearly in the final report.

## 3. Results

The thematic analysis of nursing instructors’ experiences revealed two main themes: (1) Challenges to Nursing Students’ Resilience and (2) Support Strategies for Enhancing Resilience, as presented in Table 2. Each theme is further divided into sub-themes that provide a more detailed understanding of the factors affecting nursing students’ resilience during clinical education. These themes and sub-themes align with the Unitary Caring Science Resilience-Building Model, demonstrating how nursing education naturally incorporates many of the model’s key strategies.

### 3.1. Challenges to Nursing Students’ Resilience

#### 3.1.1. Bridging Theory and Practice

Nursing instructors identified a significant challenge for students in applying theoretical knowledge to practical situations. This gap often resulted in students struggling to confidently care for patients, despite having the necessary knowledge base. As one participant noted:

*The main challenge is bridging theoretical knowledge to practical application. Students often struggle to apply the knowledge they have learned to effectively care for patients, particularly in terms of confidence.*

(P2B)

Another participant highlighted a crucial challenge in nursing education which is the gap between theoretical knowledge and practical application. She identified confidence in applying learned theory to real-world situations as a key factor in developing resilience among nursing students during clinical practice and notes that the transition from classroom to clinical setting is often problematic.

*…the second factor is confidence in their knowledge to implement and apply what they have learned theoretically into real practice. This aspect is felt to be problematic in promoting resilience among nursing students during clinical practice.*

(P4J)

#### 3.1.2. Upholding Confidence in Clinical Skills

Nursing students frequently exhibited a lack of confidence when performing nursing activities in real clinical settings. This manifested as hesitation, uncertainty, and fear, particularly when dealing with procedures involving sharp objects or direct patient care. One participant shared:

*Students may not be able to control their excitement when performing nursing activities in real situations. This may lead to them displaying uncertainty, which can affect their confidence in performing nursing activities.*

(P2I)

The lack of confidence was particularly noticeable in certain procedures, as another instructor pointed out:

*As second-year students, they are still developing nursing skills and it’s their first time practicing in clinics. Needle stick incidents occur more frequently among second-year students compared to other years. After such incidents, it affects them psychologically; they become afraid of sharp objects and lack confidence in handling sharp materials, resulting in a lack of confidence in performing nursing skills.*

(P2F)

The above quotes illuminate the transition of second-year students into clinical practice. They vibrantly portray the vulnerability of nursing students as they navigate the hands-on aspects of nursing for the first time.

#### 3.1.3. Adapting to New Clinical Environments

The transition between different clinical placements posed a challenge for students. Each new ward or specialty required students to adapt quickly to unfamiliar environments, staff, and patient populations, which could be stressful and impact their resilience. One participant explained:

*Changing wards means changing the context, for example, from a medical ward to a surgical ward, the tasks will be different, and the environment will be different. Therefore, the student’s adjustment will also differ.*

(P3A)

Another participant pointed out that, in addition to common challenges like fear, anxiety, and the pressures of first-time clinical practice, nursing students’ resilience is also influenced by their lack of familiarity with their clinical instructors and peers. She addressed how the unfamiliarity can add to the difficulties nursing students face in adapting to the clinical environment.

*Besides fears, anxieties, and the experience of their first clinical practice, another factor that affects the resilience of the nursing students is their unfamiliarity with both the clinical instructors and peers in the same group.*

(P4J)

#### 3.1.4. Managing Expectations

During clinical practice, nursing students faced various expectations from instructors, ward staff, and patients. These expectations, combined with the pressure to perform well and meet course objectives, could overwhelm students and negatively affect their resilience. One participant observed:

*As for the clinical placements, some staff might have expectations towards the students. These expectations are a challenge in promoting resilience among nursing students during clinical practice.*

(P3P)

The pressure was particularly notable for more advanced students. Another participant addressed an unexpected challenge faced by more advanced nursing students, particularly those in their fourth year. Despite their increased experience, these students can find themselves under significant stress during clinical placements.

*Another thing I’ve noticed is that when we supervise higher-year students, like fourth-year students, they may experience stress or reduced resilience. When they go for initial orientation to the placement, senior nurses may express high expectations, assuming fourth-year students should already be proficient. This can lead to added pressure.*

(P1E)

### 3.2. Support Strategies for Enhancing Resilience

#### 3.2.1. Providing Comprehensive Preparation

Participants emphasized the importance of thorough preparation before clinical placements. This included orientation sessions, skills reviews, and discussions about potential challenges students might face in specific clinical settings. One participant described her approach:

*When it’s time for clinical practice, we have a program to prepare students before they start the clinical practice. This involves discussions with coordinating instructors and designing preparation activities for nursing students, both in terms of knowledge and mental readiness.*

(P2A)

Another participant emphasized the importance of nurturing nursing students from their first year by focusing on discipline, involvement in activities, and responsibility. She highlighted the college’s role in human development, suggesting that a strong foundation of knowledge and understanding helps students build resilience throughout their educational journey.

*We all need to nurture the students from their first year, including aspects of discipline, participation in various activities, and fulfilling various responsibilities. Thus, the college’s role in fostering this is increasingly about human development. Therefore, when students have knowledge and understanding, it will help build resilience for them.*

(P4N)

#### 3.2.2. Fostering Open Communication

Creating opportunities for open dialogue between instructors and students was identified as crucial. This involved regular check-ins, post-conference sessions, and encouraging students to express their concerns and reflections freely. One participant shared her method:

*During the post-conference each day, I provide a time for the students to express their feelings, and I actively listen without making judgments. When I don’t judge, the students are more willing to share everything, like problems or stressors they encounter.*

(P3P)

Another participant exemplifies a crucial aspect of fostering open communication in clinical nursing education with patients. The instructor creates an environment of trust and autonomy by allowing nursing students to proceed without interruption during patient procedures.

*We let the students proceed without intervening, especially when they are in front of patients during procedures. This is crucial because we tell the patients that the nursing student will take care of them from the early stages of clinical practice.*

(P4J)

#### 3.2.3. Implementing Peer Support Systems

Nursing instructors found that facilitating peer support among students enhanced resilience. This included pairing students for certain activities, organizing group discussions to share experiences, and creating a supportive learning environment. One participant described her approach:

*I’ve tried pairing students who are in the same responsibility zone to assess the patient together, just for the first time. This allows students to have a peer and to share cases. When they do assignments, they still work alone, but having a buddy for the first patient interaction makes them feel more confident and less anxious.*

(P2C)

Another participant emphasized the value of group discussions as she illustrated how group discussions during post-conference sessions are used to boost morale among nursing students. After a day of clinical practice, students come together to reflect on their experiences, share their feelings, identify areas for improvement, and acknowledge their successes, fostering a supportive and open environment through conversational group dialogue.

*We use a group discussion format mainly to strengthen each other’s morale during post-conference. After a day of clinical practice, we gather to discuss what each person did, how they felt, what they want to improve or develop, or what they feel they did well. We use a conversational style in groups.*

(P1F)

#### 3.2.4. Utilizing Reflective Practice

The use of reflective activities, such as journaling, care cards, and structured debriefing sessions, was highlighted as an effective strategy to help students process their experiences and build resilience over time. One participant shared her experience with reflective notebooks:

*From my own experience, when I was a new graduate and started supervising BCPN, I provided each student with a notebook. So, we used notebooks as materials for students to write down or reflect their daily experiences. What they encountered, any worries they had, or any events of the day.*

(P1A)

Another participant described the use of a ‘Care Card’. She explained the use of a Care Card as an assessment tool for nursing students’ daily feelings during their transition between different clinical wards. She highlighted the importance of setting mutual expectations before students begin in a new ward to help them feel motivated and prepared for the changes, easing their adjustment to new environments.

*In terms of the ‘Care Card’ I mentioned earlier, it’s an assessment of students’ daily feelings. Because during the transition from the medical ward to surgical ward, or from surgical ward to medical ward, students have expectations for the new ward, which is different from the previous one. Therefore, we need to create mutual expectations beforehand to make students feel motivated for the day.*

(P3Y)

These themes and sub-themes highlight the complex interplay of factors affecting nursing students’ resilience during clinical education and the multifaceted approaches instructors use to support and enhance this crucial quality in future healthcare professionals.

## 4. Discussion

This study explored nursing instructors’ experiences regarding the challenges nursing students face in developing resilience during clinical education, as well as the strategies employed to support and enhance their resilience. The findings reveal a complex interaction of influences that impact nursing students’ resilience, organized into two main themes: (1) Challenges to Nursing Students’ Resilience and (2) Support Strategies for Enhancing Resilience. These themes highlight the critical role of nursing instructors in both identifying obstacles to resilience and implementing effective support measures among nursing students during clinical education. The results provide valuable insights into the multifaceted nature of resilience in the context of clinical education, offering a comprehensive view of both the hurdles students encounter and the strategies instructors employ to overcome them. This dual perspective offers potential paths for improving practices in nursing education, emphasizing the need for a holistic approach that addresses both the challenges and support mechanisms in fostering resilience.

The first main theme identified in this study, Challenges to Nursing Students’ Resilience, encompasses four key areas that nursing instructors perceived as significant hurdles for students in developing resilience during their clinical education. The sub-theme “Bridging Theory and Practice” emerging as a significant obstacle. Nursing instructors identified a substantial gap between theoretical knowledge and practical application, which aligns with previous research highlighting the importance of this connection for enhancing students’ resilience and academic success [24,25]. The difficulty in translating classroom learning to bedside care can decrease confidence and increase stress, potentially undermining resilience. The innovative pedagogical approaches such as problem-based learning (PBL), simulation-based education, and early clinical exposure have been suggested to address this challenge [26]. Studies have shown that PBL, in particular, is effective in developing nursing students’ clinical reasoning, competency, and problem-solving abilities, leading to improved critical thinking skills and overall satisfaction with clinical experiences [27,28,29]. The ability to flexibly adapt learned knowledge in dynamic, real-world scenarios is crucial not only for clinical skill development but also for building resilience in nursing practice [30]. This challenge of bridging theoretical understanding with practical application is critical in nurturing competent and adaptable future healthcare professionals [31]. In addressing the challenge of bridging theory and practice, nursing instructors’ experiences align with the strategy of “Inspiring and maintaining faith-hope” from the Unitary Caring Science Resilience-Building Model [17]. Nursing instructors are essentially inspiring faith and hope in students’ abilities by helping students apply theoretical knowledge in practical settings [32]. This approach can help students maintain confidence and resilience when faced with the challenges of translating classroom learning to clinical practice. Nursing instructors are essentially inspiring faith and hope in students’ abilities, a key aspect of building resilience according to the model by addressing this challenge. For example, problem-based learning and simulation-based education, as mentioned by instructors, can be seen as tools to foster this faith and hope, helping students develop the confidence necessary to navigate the complex transition from classroom to clinical setting.

The sub-theme “Upholding Confidence in Clinical Skills” emerged as another noteworthy finding. This insecurity can significantly impact students’ resilience, as it may lead to increased anxiety and stress during clinical placements. The fear associated with certain procedures, such as those involving sharp objects, highlights the need for targeted interventions to build students’ confidence in these areas. Recent studies identified fear and anxiety related to clinical procedures as significant stressors for nursing students. The psychological impact of incidents such as needlestick injuries, as mentioned by one of the instructors, can have long-lasting effects on students’ confidence and performance [33,34]. Developing structured support systems and providing ample opportunities for supervised practice could help address this challenge. Repeated exposure to clinical skills in a safe, supportive environment can significantly improve students’ confidence [35]. Additionally, a recent study suggested that peer mentoring during nursing students’ clinical placements strengthens both academic and personal support, boosts teaching and leadership abilities, and fosters student empowerment [36]. This sub-theme relates to the Unitary Caring Science Resilience-Building Model strategy of “Embracing loving-kindness for self and others”. Nursing instructors’ observations regarding students’ lack of confidence in clinical skills can be addressed through the strategy of “Embracing loving-kindness for self and others”, as proposed by the resilience-building framework [17]. This approach emphasizes the importance of self-compassion and kindness, which can help build students’ confidence and resilience. In addition, nursing instructors can promote resilience by modeling compassionate behavior and encouraging nursing students to practice self-kindness, especially when facing challenging clinical situations [37]. The structured support systems and supervised practice opportunities suggested by instructors align with this strategy, providing a supportive environment where students can build confidence through loving-kindness. Moreover, nursing instructors’ efforts to help students maintain confidence in their clinical skills reflect the model’s emphasis on self-compassion and maintaining hope in the face of challenges.

The sub-theme “Adapting to New Clinical Environments” presents another challenge to students’ resilience. This constant need for adaptation can be mentally and emotionally taxing, potentially leading to burnout if not properly managed. The findings suggest that the unfamiliarity extends beyond the physical environment to include interpersonal relationships with staff and peers. This aligns with the concept of reality shock which nursing students may experience repeatedly with each new clinical placement [38]. The stress of adapting to new environments can be particularly challenging for nursing students, as it requires them to navigate complex social dynamics while also focusing on their learning objectives [39]. Developing strategies to ease these transitions and help nursing students quickly adapt to new environments could be crucial in maintaining their resilience throughout their education. Orientation programs that focus not only on the physical layout and procedures of a new ward but also on the social and cultural aspects of the environment could be beneficial [40]. Additionally, fostering a sense of belonging and social support within each new clinical setting has been shown to enhance student resilience and performance [41]. The challenge of adapting to new clinical environments, as identified by nursing instructors, corresponds well with the Unitary Caring Science Resilience-Building Model strategy of “Deepening a creative use of self and sense of belonging” in our adopted theoretical model [17]. The study’s findings highlighted how nursing instructors foster the creative adaptability and sense of belonging that the Unitary Caring Science model identifies as crucial for resilience by helping students navigate these transitions. Nursing instructors can support nursing students in this process by creating welcoming atmospheres and helping students value their unique experiences in each new environment [42]. The suggestion of orientation programs that focus on the social and cultural aspects of new clinical settings aligns with this strategy, fostering a sense of belonging that can enhance students’ resilience and adaptability.

The sub-theme “Managing Expectations and Pressures” emerged as a significant challenge to students’ resilience. This finding is consistent with recent research that identified perceived expectations as a major source of stress for nursing students [43,44]. The added pressure on more advanced students to perform at a higher level further compounds this challenge. These expectations, while often well-intentioned, can create a high-stress environment that may be counterproductive to learning and resilience-building [31]. This suggests a need for a more balanced approach to setting expectations and providing support throughout the nursing education journey. Moreover, recent research found that nursing students often experience impostor feelings, which can negatively impact their confidence and performance [45]. Addressing these feelings and helping students develop a realistic self-assessment of their abilities could be crucial in building resilience. In addressing the challenge of managing various expectations and pressures, nursing instructors’ experiences align with the Unitary Caring Science Resilience-Building Model strategy of “Valuing forgiveness and releasing negativity” from the resilience-building framework [17]. This strategy involves cognitive reframing and shifting focus, which can help students cope with the pressures they face. The study’s findings addressed how nursing instructors’ efforts can help students manage these expectations that align with the model’s emphasis on reframing negative experiences and maintaining a positive outlook. Nursing instructors can support this by teaching students techniques for reframing situations and encouraging a growth mindset [42]. This approach can help students manage the expectations from various sources and maintain resilience in the face of challenging situations.

The second main theme identified in this study, Support Strategies for Enhancing Resilience, highlights the proactive measures nursing instructors employ to foster resilience among nursing students during clinical education. These strategies, as reported by the instructors, are crucial in helping students navigate the challenges they face and develop the resilience necessary for a successful nursing career. The sub-theme “Providing Comprehensive Preparation” perceived by nursing instructors emphasized the importance of thorough preparation before clinical placements as a key strategy for enhancing student resilience. This approach aligns with previous research highlighting the positive impact of preparatory interventions on nursing students’ clinical performance and stress reduction [46]. The nursing instructors’ focus on both knowledge and mental readiness demonstrates a holistic approach to preparation, addressing not only the cognitive aspects but also the psychological demands of clinical practice [47]. The practice of providing detailed ward-specific information is particularly noteworthy. This strategy can help reduce the anxiety associated with entering new clinical environments, a challenge identified in the first main theme. This comprehensive preparation strategy aligns with the “Embracing loving-kindness for self and others” approach from the Unitary Caring Science Resilience-Building Model [17]. Nursing instructors demonstrate loving-kindness towards their students, helping them feel cared for and supported as they enter challenging clinical environments by providing thorough preparation [48]. This approach can significantly enhance students’ resilience by reducing anxiety and increasing confidence. Moreover, nursing instructors are effectively bridging the gap between classroom learning and clinical practice, thus addressing the theory-practice gap identified earlier by familiarizing students with potential cases and required skills beforehand [49].

The sub-theme “Fostering Open Communication”, which emphasizes creating opportunities for candid interactions between instructors and students, emerged as a crucial support strategy. This approach is consistent with research underscoring the importance of effective communication in reducing stress and enhancing resilience among nursing students [47]. The use of post-conference sessions and non-judgmental listening, as described by the instructors, creates a safe space for students to express their concerns and reflect on their experiences. Particularly interesting is the strategy of allowing students to proceed without intervention during patient procedures, as mentioned by one instructor. This approach not only fosters open communication with patients but also builds students’ confidence and autonomy, addressing the lack of confidence in clinical skills identified as a challenge in the first theme. This strategy resonates with the Unitary Caring Science Resilience-Building Model strategy of “Nurturing interpersonal and intersubjective connections/relations” [17]. Nursing instructors are creating opportunities for meaningful connections and promoting a caring-healing work environment, which are crucial elements in building resilience by fostering open communication. This practice exemplifies the model’s emphasis on creating trusting relationships to cope with stress and build resilience.

The sub-theme “Implementing Peer Support Systems”, which highlights facilitating peer support among students as a means of enhancing resilience, is well-supported by current literature. Peer support has been shown to be an effective strategy for reducing stress and improving academic performance among nursing students [50]. The practice of pairing students for initial patient interactions, as described by one instructor, is a novel approach that merits further investigation. The practice of facilitating group discussions to strengthen morale and share experiences aligns with the concept of collective resilience, where group solidarity and shared social identity contribute to increased resilience among nursing students [12]. This approach not only helps students process their experiences but also creates a supportive learning environment that can buffer against the stressors of clinical education. This peer support strategy corresponds well with both “Nurturing interpersonal and intersubjective connections/relations” and “Deepening a creative use of self and sense of belonging”, as outlined in our theoretical framework [17]. Nursing instructors are creating opportunities for meaningful connections among students and promoting a sense of belonging within the clinical education environment by fostering peer support systems [51]. These interpersonal connections and the feeling of belonging can significantly enhance resilience by providing emotional support and a shared sense of purpose among nursing students. Moreover, the peer support systems foster the interpersonal connections and sense of belonging that the Unitary Caring Science model identifies as crucial for resilience.

The sub-theme “Utilizing Reflective Practice” addresses the use of reflective activities, such as journaling and structured debriefing sessions, which emerged as a key strategy for building resilience over time. This approach is well-supported by research demonstrating the positive impact of reflective practice on nursing students’ critical thinking skills and emotional intelligence [30]. The use of reflective notebooks and ‘Care Cards’, as described by the instructors, provides concrete tools for implementing this strategy. Particularly noteworthy is the use of the ‘Care Card’ to assess students’ daily feelings and create mutual expectations during ward transitions. This practice directly addresses the challenge of adapting to new clinical environments identified in the first theme, providing a structured approach to managing the stress associated with these transitions. This reflective practice strategy aligns closely with the “Balancing self-learning, self-awareness, and an evolved consciousness” approach from the Unitary Caring Science Resilience-Building Model [17]. Nursing instructors are fostering self-awareness and promoting an evolved consciousness that can significantly enhance resilience by encouraging students to reflect on their experiences and emotions. Nursing instructors’ experiences reveal both the challenges faced by nursing students and the innovative strategies employed to foster resilience in the clinical setting. These reflective practices directly support our adopted model’s emphasis on self-learning and awareness as key components of resilience.

Nursing instructors can more effectively support students in overcoming the challenges they face during clinical education by integrating these resilience-building strategies into their teaching and mentoring approaches. Additionally, the support strategies demonstrate a comprehensive and multifaceted approach to enhancing nursing students’ resilience during clinical education. Nursing instructors are addressing the challenges identified in the first theme and creating an environment conducive to resilience-building by providing comprehensive preparation, fostering open communication, implementing peer support systems, and utilizing reflective practice. These strategies, grounded in the experiences of nursing instructors and aligned with the Unitary Caring Science Resilience-Building Model, offer valuable insights for improving nursing education practices and supporting the development of resilient future healthcare professionals.

### 4.1. Implications for Nursing Education and Practice

The findings of this study have significant implications for nursing education and practice, particularly in enhancing students’ resilience during clinical education. A primary focus should be on bridging theory and practice through innovative pedagogical approaches such as problem-based learning, simulation-based education, and early clinical exposure. These methods, combined with increased opportunities for supervised practice of clinical skills and peer mentoring programs, can help build students’ confidence and adaptability in various healthcare settings. Additionally, nursing education programs should prioritize comprehensive preparation before clinical placements, addressing knowledge-based and psychological readiness and providing detailed, ward-specific information to reduce anxiety and enhance resilience.

Creating a supportive learning environment is crucial for fostering resilience. This includes developing orientation programs that address both physical and socio-cultural aspects of new clinical environments, setting realistic expectations for students at different levels, and creating regular opportunities for open dialogue. Formal integration of peer support systems and reflective practices, such as journaling and structured debriefing sessions, can further enhance students’ self-awareness and emotional intelligence. A holistic approach to resilience-building, drawing on frameworks like the Unitary Caring Science Resilience-Building Model, can address the cognitive, emotional, and interpersonal aspects of resilience throughout nursing education. Lastly, continuous professional development for nursing instructors on resilience-building strategies is essential for effective implementation in clinical education settings.

### 4.2. Limitations and Future Research

This study offers insights into nursing students’ resilience during clinical education, yet several limitations should be acknowledged. The participant sample was restricted to nursing instructors from four nursing colleges within a single educational system in Thailand, potentially limiting the transferability of findings to other contexts. The perspectives captured represent only those of instructors, without including the valuable viewpoints of nursing students themselves. Additionally, the cross-sectional nature of the study provides only a momentary understanding of resilience development rather than capturing its evolution over time. Future research might benefit from expanding participant diversity across varied institutional settings and geographical regions. Including nursing students’ perspectives would enrich understanding of resilience experiences from multiple viewpoints. A longitudinal approach could illuminate how resilience develops throughout educational progression and early professional practice, providing deeper insights into the sustainability of resilience-building strategies. The integration of quantitative resilience measures alongside qualitative approaches might strengthen the evidence base for intervention effectiveness. Researchers could focus on developing and evaluating targeted interventions based on the strategies identified in this study, such as structured peer support programs or reflective practice frameworks. Exploring cultural factors influencing resilience development appears particularly relevant in increasingly diverse healthcare education environments. As digital technologies become more prevalent in education, investigating technology-enhanced approaches to resilience-building represents an important area for exploration. Examining resilience development within interprofessional educational contexts might address the collaborative demands of contemporary healthcare delivery. Assessing how resilience-building strategies impact professional outcomes including career satisfaction and burnout prevention could establish the practical value of educational interventions. Further refinement of theoretical frameworks, such as the Unitary Caring Science Resilience-Building Model, might facilitate more contextually appropriate approaches to nursing education. Through careful attention to these research directions, the understanding of resilience development among nursing students may be enhanced, potentially supporting the preparation of healthcare professionals equipped for the challenges of clinical practice.

## 5. Conclusions

This study highlights the complex challenges nursing students face in developing resilience during clinical education and the multifaceted strategies instructors employ to support this process. Nursing education can foster more resilient future healthcare professionals by addressing the theory–practice gap, building confidence in clinical skills, easing transitions between clinical environments, and managing expectations, while also implementing comprehensive preparation, open communication, peer support, and reflective practices. The findings underscore the need for a holistic approach to nursing education that considers not only clinical competence but also the psychological well-being and resilience of students. This approach requires a collaborative effort from educational institutions, clinical placement sites, instructors, and students themselves.

As the healthcare landscape continues to evolve and present new challenges, the ability to cultivate resilience in nursing students becomes increasingly crucial. In addition, nursing education can better prepare the next generation of healthcare professionals to thrive in complex and demanding healthcare environments by implementing evidence-based strategies to support and enhance student resilience. These findings provide a foundation for developing more effective approaches to nursing education that prioritize not only the clinical competence but also the psychological well-being and resilience of nursing students. As we move forward, continued research and innovation in this area will be essential to ensure that nursing education evolves to meet the changing needs of students and the healthcare system.

Moreover, the analysis through the lens of the Unitary Caring Science resilience-building model reveals that nursing education naturally incorporates many of its key strategies. The challenges faced by nursing students and the support strategies employed by nursing instructors align closely with the model’s principles, suggesting that this framework could provide valuable insights for further developing and refining approaches to building resilience in nursing education.

## Figures and Tables

**Table 1:** Focus group interview guide.

Type of Questions	Example of Questions
Greetings/General information/ Introductory questions	Hello, and welcome to our focus group. How is everyone doing today? May I ask, how old is each of you? How many years have each of you had the experience of clinical supervising nursing students in their first clinical practice? What clinical placement did each of you supervise nursing students in your last clinical supervision on their first clinical practice?
Engagement questions	Today, we will be discussing experiences with challenges and supports to promote nursing students’ resilience during clinical supervision of their first clinical practice. What thoughts or feelings does each of you have when you think about promoting resilience in nursing students during the first clinical practice? Could each of you share a story about how you have encountered with promoting resilience among nursing students during the first clinical practice, please?
Exploratory questions	What is the biggest challenge to promote nursing students’ resilience during the first clinical practice? What is the first thing that comes to mind when thinking about support to promote nursing students’ resilience during the first clinical practice? Is there any support provided by the college that helps promote resilience among nursing students? If so, please tell me more about it.
Follow-up questions	Who has had a similar experience to the last informant? Who has had a different experience from the last informant? Is there anyone that want to add something about this point? Could you explain a bit more about this point?
Exit questions	Thank you for your time. Before we wrap up, is there anything that you would like to mention? Do any of you think there is something important that we missed in our discussion today?

**Table 2:** Themes and subthemes.

Main Themes	Sub-Themes
Challenges to Nursing Students’ Resilience	Bridging Theory and Practice Upholding Confidence in Clinical Skills Adapting to New Clinical Environments Managing Expectations
Support Strategies for Enhancing Resilience	Providing Comprehensive Preparation Fostering Open Communication Implementing Peer Support Systems Utilizing Reflective Practice

## Data Availability

The datasets used and analyzed during the current study are available from the corresponding author on reasonable request.

## Public Involvement Statement

No public involvement in any aspect of this research.

## Guidelines and Standards Statement

This manuscript was drafted against the Consolidated criteria for reporting qualitative research (COREQ): for qualitative research. (https://www.equator-network.org/reporting-guidelines/coreq/ accessed on 9 January 2025).
